# Bayesian analysis of a systematic review of early *versus* late tracheostomy in ICU patients

**DOI:** 10.1016/j.bja.2022.08.012

**Published:** 2022-09-24

**Authors:** Laura Quinn, Tonny Veenith, Julian Bion, Karla Hemming, Tony Whitehouse, Richard Lilford

**Affiliations:** 1 Institute of Applied Health Research, University of Birmingham, Birmingham, UK; 2 NIHR Birmingham Biomedical Research Centre, University Hospitals Birmingham NHS Foundation Trust and University of Birmingham, Birmingham, UK; 3 Department of Critical Care and Anaesthesia, Queen Elizabeth Hospital Birmingham, Birmingham, UK; 4 Intensive Care Medicine, University of Birmingham, Birmingham, UK

**Keywords:** Bayesian meta-analysis, early tracheostomy, intensive care unit, mechanical ventilation, mortality, respiratory failure, ventilator-associated pneumonia

## Abstract

**Background:**

A recent systematic review and meta-analysis of RCTs of early *vs* late tracheostomy in mechanically ventilated patients suggest that early tracheostomy reduces the duration of ICU stay and mechanical ventilation, but does not reduce short-term mortality or ventilator-associated pneumonia (VAP). Meta-analysis of randomised trials is typically performed using a frequentist approach, and although reporting confidence intervals, interpretation is usually based on statistical significance. To provide a robust basis for clinical decision-making, we completed the search used from the previous review and analysed the data using Bayesian methods to estimate posterior probabilities of the effect of early tracheostomy on clinical outcomes.

**Methods:**

The search was completed for RCTS comparing early vs late tracheostomy in the databases PubMed, EMBASE, and Cochrane library in June 2022. Effect estimates and 95% confidence intervals were calculated for the outcomes short-term mortality, VAP, duration of ICU stay, and mechanical ventilation. A Bayesian meta-analysis was performed with uninformative priors. Risk ratios (RRs) and standardised mean differences (SMDs) with 95% credible intervals were reported alongside posterior probabilities for any benefit (RR<1; SMD<0), a small benefit (number needed to treat, 200; SMD<–0.5), or modest benefit (number needed to treat, 100; SMD<–1).

**Results:**

Nineteen RCTs with 3508 patients were included. Comparing patients with early *vs* late tracheostomy, the posterior probabilities for any benefit, small benefit, and modest benefit, respectively, were: 99%, 99%, and 99% for short-term mortality; 94%, 78%, and 51% for VAP; 97%, 43%, and 1% for duration of mechanical ventilation; and 97%, 75%, and 27% and for length of ICU stay.

**Conclusions:**

Bayesian meta-analysis suggests a high probability that early tracheostomy compared with delayed tracheostomy has at least some benefit across all clinical outcomes considered.

**Editor's key points**

• A previous systematic review using frequentist methods concluded that early tracheostomy was more effective in reducing length of ICU stay and duration of mechanical ventilation but not short-term mortality or ventilator-associated pneumonia.
• This Bayesian analysis provides evidence that early tracheostomy improves all of the above outcomes.
• Investigators should provide probabilities of effect sizes rather than simply dichotomise outcomes based on a hypothesis test.

Tracheostomy is a common surgical procedure performed in critically ill patients who require prolonged mechanical ventilation.^1^ Tracheostomy is hypothesised to reduce ventilator-associated pneumonia (VAP), duration of mechanical ventilation, length of intensive care unit (ICU) stay, and risk of death,^2^ in part through permitting a reduction in sedation, improving patient comfort, and facilitating communication. However, tracheostomy is associated with bleeding, wound infection, tracheal stenosis, accidental displacement, and occasionally death.^3^

The timing of tracheostomy in a mechanically ventilated patient is a clinically important question in ICU practice; several studies have attempted to answer this, including several meta-analyses.^2^, ^3^, ^4^, ^5^, ^6^ Deng and colleagues^4^ completed a recent meta-analysis based on RCTs to investigate whether early tracheostomy compared with late tracheostomy can improve clinical outcomes in critically ill patients undergoing mechanical ventilation. This study concluded that early tracheostomy reduces the length of ICU stay and mechanical ventilation but does not reduce the risk of short-term mortality or VAP. Although the effects sizes for short-term mortality (risk ratio [RR]=0.87; 95% confidence interval [CI], 0.74–1.03) and VAP (RR=0.90; 95% CI, 0.78–1.04) suggested a reduction in risk, the results were not statistically significant at the 5% level.

The above analysis used frequentist methods by dichotomising results into just ‘null’ or positive.^7^ However, we think it would be more of use to present clinical decision-makers with the probability of the intervention on the magnitude of a particular outcome. Such an approach requires a Bayesian calculation of ‘posterior probabilities’. To obtain a source of data for calculation of these probabilities, we completed a search using the strategy described by Deng and colleagues in their previous systematic review.^7^ We then analysed the data using standard frequentist methods followed by a Bayesian meta-analysis. We estimated the probability of any benefit, a small benefit (defined as a number needed to treat [NNT] of 200 for binary outcomes or standardised mean difference [SMD] <–0.5 for continuous outcomes) and a modest benefit (defined as an NNT of 100 or SMD <–1.0).

## Methods

### Search strategy and selection criteria

Our analysis is based on a previous review and frequentist meta-analysis described by Deng and colleagues^4^ comparing the risk of clinical outcomes in ICU patients who had ‘early’ tracheostomy *vs* ‘late’ tracheostomy. This review was carried out following the Preferred Reporting Items for Systematic Reviews and Meta-Analysis recommendations. The databases PubMed, EMBASE, and the Cochrane library were searched systematically in June 2022 using the keywords ‘tracheotomy’ OR ‘tracheostomy’ AND ‘mechanical ventilation’ OR ‘intracheal intubation’ AND ‘randomised controlled trials’. The search was independently performed by two reviewers, and discrepancies were resolved by a third observer. The inclusion criterion was RCTs with a population of ICU patients requiring mechanical ventilation, an intervention group with ‘early’ tracheostomy, a comparison group with ‘late’ tracheostomy, and one of the following clinical outcomes: short-term mortality, VAP, length of ICU stay, duration of mechanical ventilation, and length of hospital stay. Another inclusion criterion was use of an intention-to-treat analysis. Some studies clearly stated this approach, whereas others did not include a specific statement to this effect. We included these studies in our review unless there was some indication in the numbers that such an approach had not been followed. For example we excluded papers where there was wide disparity in control and intervention participants despite a 1:1 randomisation ratio.

### Data extraction for this review

Information on the study setting, population, timing of early and late tracheostomy, and clinical outcomes was extracted. We excluded one of the outcomes in the review of Deng and colleagues, length of hospital stay, on the ground that only six studies in the review included this outcome. For the binary outcomes (short-term mortality and VAP), we extracted the number of times a clinical outcome occurred and the number of patients in the early and late tracheostomy groups. For the continuous outcomes (length of ICU stay and duration of mechanical ventilation), we extracted means and standard deviations in both groups or medians and inter-quartile ranges when the mean and standard deviation were not reported.

### Statistical methods

A Bayesian random effects meta-analysis was performed to account for between trial variation in treatment effects, and variability within a trial. Heterogeneity in the effect of early tracheostomy on clinical outcomes across the studies was measured using tau and a value of more than one was considered ‘fairly extreme’.^8^ Posterior probabilities were calculated for the heterogeneity parameters and plots of the distribution of the probabilities were created.

#### Binary outcomes

For binary outcomes, the number of times a clinical outcome occurred and the number of patients in the early and late tracheostomy groups were used to calculate the RRs and standard errors. For the analysis of the binary outcomes, uninformative priors were used to minimise assumptions about the model, including *N* (0, 1000) prior for the effect parameter and a uniform prior for the heterogeneity parameter, and the data were pooled on the log-scale to estimate for the distribution of the log RRs. The pooled log RRs were transformed through exponentiation to calculate RRs. RRs with their 95% credible intervals (95% CrL) were reported, and plots of the posterior distribution of the log RRs were created. In addition, we report posterior probabilities for any benefit (RR<1); a small beneficial effect defined as an absolute risk difference of at least 0.5% (equivalent to a number needed to treat [NNT] ≤200); and a modest beneficial effect defined as a risk difference of at least 1% (NNT ≤100). The risk differences of 0.5% and 1% were chosen as they was used in a previous Bayesian re-analysis,^9^ and it seemed plausible that patients and their families would be willing to offset the disadvantages of tracheostomy against potential reductions in mortality. To estimate the posterior probability of these risk differences, we extracted the risk of the outcome under the control arm from the largest studies with available data in the previous systematic review; risk of short-term mortality was 31.5% in the study of Young and colleagues,^10^ and risk of VAP was 9% in the study of Diaz-Prieto and colleagues.^11^ We then calculated the equivalent log RR for each of the two putative absolute risk differences postulated above (0.5% and 1%). From the pooled distribution of the log relative risk, we then calculated the posterior probability of these two log RRs.

#### Continuous outcomes

For continuous outcomes, means, and standard deviations were used to calculate SMDs. When standard deviations were not reported, standard errors or inter-quartile ranges were extracted and converted to standard deviations. The SMDs were then calculated using Cohen's *d*. For the analysis of the continuous outcomes (duration of mechanical ventilation and ICU stay), uninformative priors were again used, including *N* (0, 1000) prior for the effect parameter and a uniform prior for the heterogeneity parameter which provided a pooled estimate for the distribution of the SMDs. The SMDs and 95% CrLs were reported. Plots of the posterior distribution of the SMDs were created. In addition, we report posterior probabilities for any benefit (defined as a SMD <0), a small beneficial effect (which we have defined as a SMD <–0.5), and a modest beneficial effect (which we have defined as a SMD <–1.0). The small and modest benefits defined here are different to the standard definition of small, medium, and large SMDs used by Cohen.^12^ To estimate the posterior probabilities on a clinically meaningful scale, we extracted the standard deviation of the outcome for the control group from the largest study with available data in the previous review by Young and colleagues.^10^ We then multiplied the SMDs by the standard deviation of the outcome in the control group standard deviation to get an estimate of the mean difference (MD) in days.

#### Frequentist method for comparison

A frequentist meta-analysis was performed for all of the clinical outcomes. RRs for the binary outcomes and SMDs for the continuous outcomes were pooled using a random-effects model. Estimates with 95% confidence intervals were reported.

#### All outcomes

All analysis was carried out using the ‘bayesmeta’ work package from RStudio v1.4 (RStudio, Inc., Boston, MA, USA).

## Results

**Fig 1:**
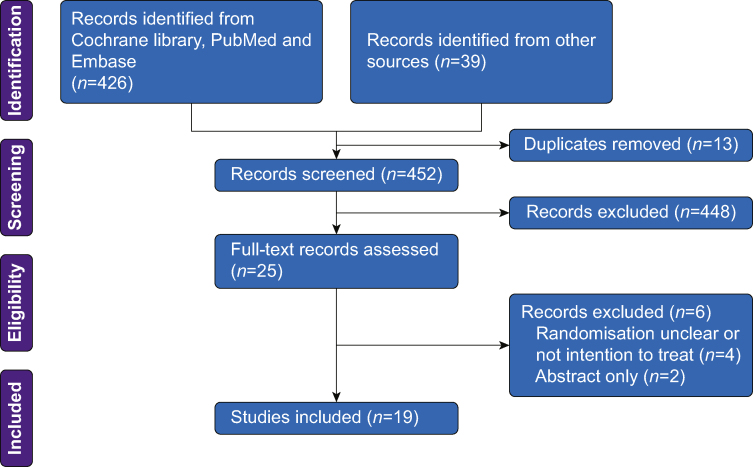
Flow diagram for search (completed June 2022).

**Fig 2:**
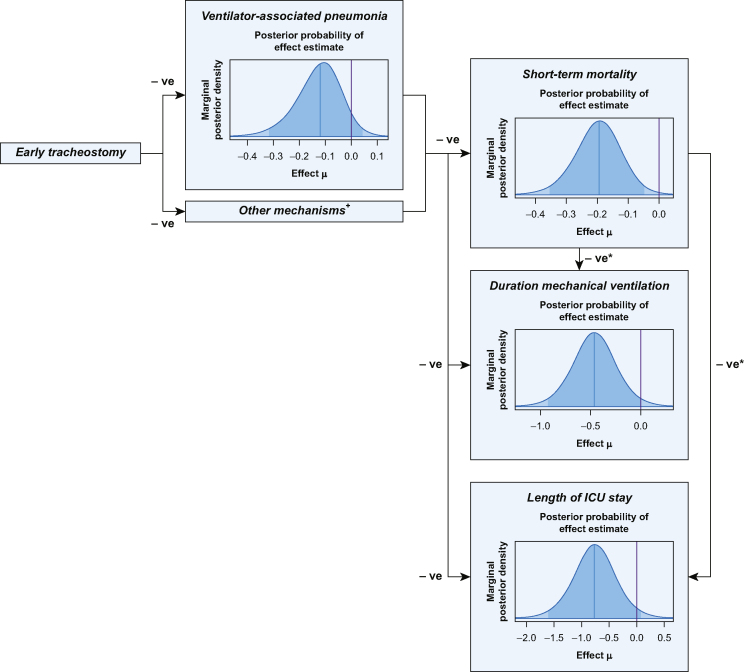
Casual pathway of clinical outcomes comparing early tracheostomy to late tracheostomy, including posterior probabilities plots of effect sizes. Posterior probability of log relative risks for ventilator-associated pneumonia and short-term mortality and standardised mean differences for duration of mechanical ventilation and length of ICU stay. Vertical line represents no difference with an effect size of zero. ⁺Other mechanisms include reduction in sedation dose, earlier mobilisation and possibly improved mucociliary action which generally have not been reported in the studies included in our review. –ve represents reduced probability of outcome. –ve∗ represents reduced probability of outcome owing to competing risks.

In total, 19 RCTs encompassing 3508 patients were included in the systematic review (Fig. 1). Baseline characteristics of the included studies are reported in Table 1. A summary of the estimated effect sizes, heterogeneity parameters, and posterior probabilities for beneficial effects and extreme heterogeneity are reported for each outcome in Table 2. For each clinical outcome, the plots of the posterior distribution for effect parameters (Fig. 2) and for heterogeneity parameters were reported (see Supplementary material Figs. 1–4).

**Table 1:** Baseline characteristics of studies included in the systematic review and meta-analysis. APACHE II, Acute Physiology and Chronic Health Evaluation II; SAPS II, Simplified Acute Physiology Score II; IQR, inter-quartile range. ∗SAPS severity scoring system. Mean (standard deviation). ^†^SAPS II severity scoring system. Mean (standard deviation). ^‡^SAPs II severity scoring system. Median [range]. ^¶^APACHE II score. Median and IQR. ^§^Glasgow Coma Score. Mean (standard deviation). ^||^Glasgow Coma Score. Median and IQR.

Study (first author, year)	Country	Setting	Timing of tracheostomy		Sample size		Disease severity (APACHE II)	
			Early	Late	Early	Late	Early	Late
Rodriguez,^13^ 1990	USA	Surgical ICU	≤7	≥8	51	55	10 (1)	10 (1)
Saffle,^14^ 2002	USA	Burn ICU	2–3	≥14	21	23	NR	NR
Bouderka,^15^ 2004	Morocco	Trauma ICU	5–6	Prolonged intubation	31	31	5 (2)∗	6 (4)∗
Rumbak,^16^ 2004	USA	Medical ICU	≤2	14–16	60	60	27 (4)	26 (3)
Barquist,^17^ 2006	USA	Trauma ICU	≤7	≥29	29	31	12 (3)	13 (5)
Blot,^18^ 2008	France	Surgical ICU	≤4	Prolonged intubation	61	62	50 [17–103]^†^	50 [15–96]^†^
Terragni,^19^ 2010	Italy	ICU	6–8	13–15	209	210	51 (9)^‡^	50 (9)^‡^
Trouillet,^20^ 2011	France	Postcardiac ICU	≤5	Prolonged intubation	109	107	47 (12)^‡^	46 (11)^‡^
Koch,^21^ 2012	Germany	Surgical ICU	≤4	≥6	50	50	21 (12–31)^¶^	22 (10–33)^¶^
Zheng,^22^ 2012	China	Surgical ICU	3	15	58	61	20 (2)	20 (3)
Bösel,^23^ 2013	Germany	Surgical ICU	≤3	7–14	30	30	17 (13–19)^¶^	16 (11–19^¶^
Young,^10^ 2013	UK	General and postcardiac ICU	≤4	≥10	451	448	20 (7)	20 (6)
Diaz-Prieto,^11^ 2014	Spain	ICU	<8	>14	245	244	20 (5–40)^¶^	19 (4–38)^¶`^
Mohamed,^24^ 2014	Egypt	ICU	<10	≥10	20	20	23 (7)	24 (8)
Filaire,^25^ 2014	France	ICU	1	Prolonged intubation	39	39	NR	NR
Karlovic,^26^ 2018	Bosnia and Herzegovina	ICU	2–4	≥15	38	42	24 (8)	22 (7)
Goo,^27^ 2022	Malaysia	Neurosurgical ICU	<7	≥7	20	19	8 (4)^§^	8 (4)^§^
Bosel,^28^ 2022	USA and Germany	Neurocritical care centres	≤5	≥10	188	194	7 (4–9)^||^	6 (3–9)^||^
Olofsson,^29^ 2022	Sweden	ICU	≤7	≥10	72	78	51 (8)∗	51 (7)∗

**Table 2:** Estimated effect sizes for clinical outcomes comparing early tracheostomy *vs* late tracheostomy and posterior probabilities for beneficial effects and extreme heterogeneity. 95% CrL, 95% credible intervals; NNT, number needed to treat; RR, risk ratios; SMD, standardised mean difference.

	Number of studies	Effect size (95% CrL)	Heterogeneity parameter – median tau	Posterior probabilities			
				Any benefit	Small benefit	Modest benefit	Extreme heterogeneity֒
**Binary outcomes**		**RR**		**RR<1**	**NNT≤200**	**NNT≤100**	**tau>1**
Short term mortality	18	0.82 (0.70 to 0.96)	0.13	99%	99%	99%	0%
Ventilator-associated pneumonia	16	0.89 (0.73 to 1.05)	0.23	94%	78%	51%	0%
**Continuous outcomes**		**SMD**		**SMD<0**	**SMD<–0.5**	**SMD<–1.0**	**tau>1**
Duration of ventilation	13	–0.46 (–0.93 to –0.01)	0.71	97%	43%	1%	11%
Length of ICU stay	12	–0.76 (–1.61 to 0.07)	1.25	97%	75%	27%	82%

**Fig 3:**
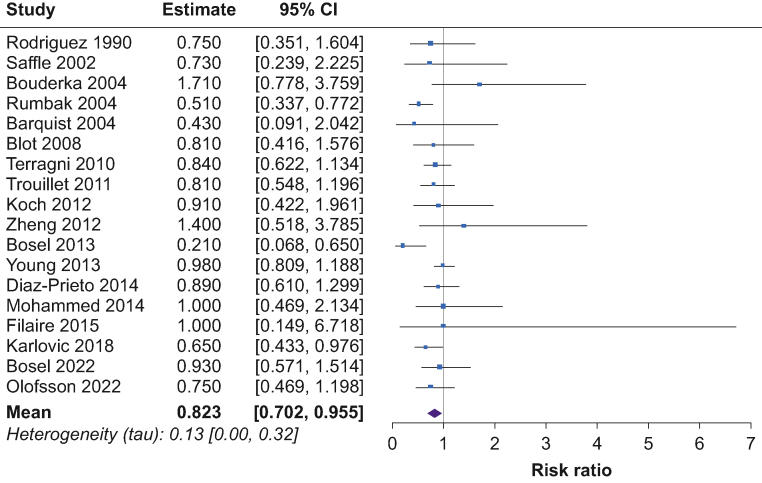
Forest plot of estimated risk ratio comparing risk of short-term mortality in early tracheostomy vs late tracheostomy patients. CI, confidence interval.

**Fig 4:**
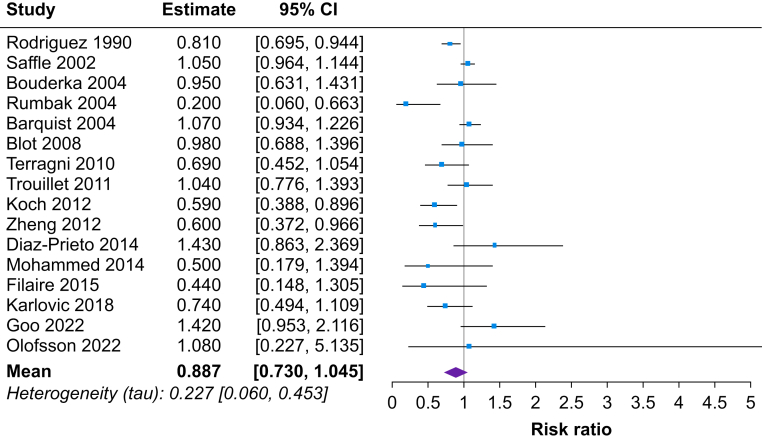
Forest plot of estimated risk ratio comparing risk of ventilator-associated pneumonia in early tracheostomy vs late tracheostomy patients. CI, confidence interval.

**Fig 5:**
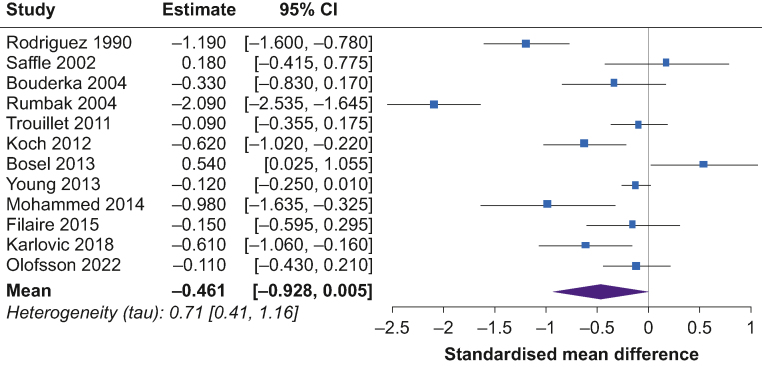
Forest plot of estimated standardised mean difference comparing duration of mechanical ventilation in early tracheostomy vs late tracheostomy patients. CI, confidence interval.

**Fig 6:**
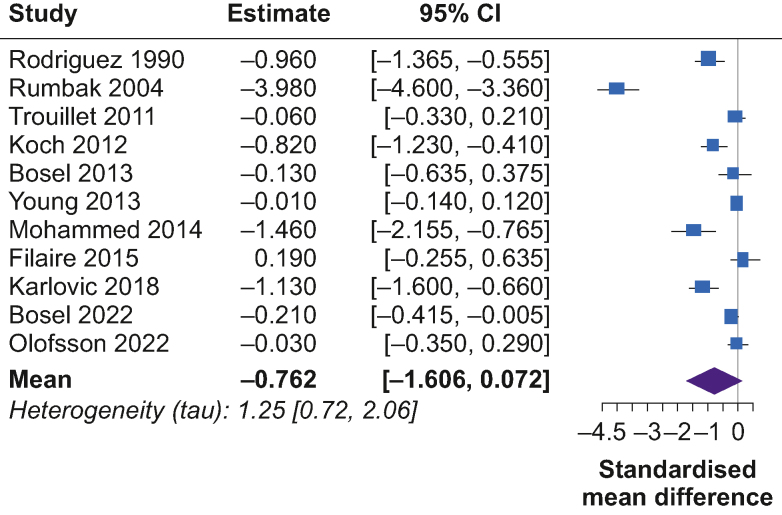
Forest plot of estimated standardised mean difference comparing duration of ICU stay in early tracheostomy vs late tracheostomy patients. CI, confidence interval.

### Short-term mortality

The pooled RR for short-term mortality was 0.82 (95% CrL, 0.70 to 0.96) for patients with early tracheostomy compared with late tracheostomy (Fig. 3). The posterior probability of any beneficial effect (RR<1) was 99%, a small beneficial effect (NNT≤200) was 99%, and a modest beneficial effect (NNT≤100) was 99%. The posterior probability for extreme heterogeneity (tau>1) in the effect of early tracheostomy on short-term mortality across the studies was 0%.

### Ventilator-associated pneumonia

The pooled mean RR for VAP was 0.89 (95% CrL, 0.73 to 1.05) for patients with early tracheostomy compared with late tracheostomy (Fig. 4). The posterior probability of any beneficial effect (RR<1) was 94%, a small beneficial effect (NNT≤200) was 78%, and a modest beneficial effect (NNT≤100) was 51%. The posterior probability for extreme heterogeneity (tau>1) was 0%.

### Duration of mechanical ventilation

The pooled SMD for the duration of mechanical ventilation was –0.46 (95% CrL, –0.93 to –0.01; MD, –7 days) for patients with early tracheostomy compared with late tracheostomy (Fig. 5). The posterior probability of any beneficial effect (SMD<0 or a mean difference <0 day) was 97%, a small beneficial effect (SMD<–0.5 or a MD<–7 days) was 43%, and a modest beneficial effect (SMD<–1 or a MD<–14 days) was 1%. The posterior probability for extreme heterogeneity (tau>1) was 11%.

### Length of ICU stay

The pooled SMD for the length of ICU stay was –0.76 (95% CrL, –1.61 to –0.07; MD, –9 days) for patients with early tracheostomy compared with late tracheostomy (Fig. 6). The posterior probability of any beneficial effect (SMD<0 or a MD<0 days) was 97%, a small beneficial effect (SMD<–0.5 or a MD<–6 days) was 75%, and a modest beneficial effect (SMD<–1 or a MD<–12 days) was 27%. The posterior probability for extreme heterogeneity (tau>1) was 82%.

### Frequentist method for comparison

Frequentist analysis was completed for each of the clinical outcomes instead of a Bayesian approach for comparison. The pooled RR was 0.82 (95% CI, 0.72 to 0.94) for short-term mortality and 0.90 (95% CI, 0.78 to 1.02) for VAP when comparing patients with early tracheostomy to late tracheostomy. The pooled SMD was –0.46 (95% CI, –0.79 to –0.13) for duration of mechanical stay and –0.73 (95% CI, –1.17 to –0.30) for length of ICU stay when comparing patients with early tracheostomy to late tracheostomy. Further information on the frequentist analysis is reported in the Supplementary material.

### Interpretation

#### Causal pathway

In Figure 2, we represent a plausible causal pathway whereby early tracheostomy might improve patient outcomes relative to delayed tracheostomy. According to this model, the relationship between early tracheostomy and short-term mortality is mediated by a reduction in VAP. Likewise, a reduction in sedation used to facilitate orotracheal tube tolerance might improve mucociliary function, communication and early mobilisation resulting in a reduction in short-term mortality, duration of mechanical ventilation, and length of ICU stay.^30^, ^31^, ^32^ In Figure 2, we have explored this possible casual pathway, which we have populated with credible limits calculated from our Bayesian re-analysis of the data in this review. The pattern in the data provides support for the theory that early tracheostomy leads to beneficial outcomes and that reduction in pneumonia is an important mediating variable. This does not mean that this is the only pathway, but we cannot test for the additional putative pathway relating to communication, early mobilisation, and mucociliary function because the salient data were not collected in the studies on which we rely, perhaps because the authors think that pneumonia is the most important variable linking intervention to outcome.

## Discussion

This Bayesian meta-analysis assessed whether early tracheostomy compared with late tracheostomy can improve clinical outcomes in critically ill patients undergoing mechanical ventilation. The results suggest that early tracheostomy has a beneficial effect on clinical outcomes. The pattern in the data is consistent with a plausible theoretical model or causal pathway.

### Comparison with previous results: binary outcomes

A meta-analysis by Deng and colleagues^4^ examined whether early tracheostomy compared with late tracheostomy can improve clinical outcomes using a frequentist approach. The review concluded that ‘early tracheotomy can reduce the length of ICU stay and mechanical ventilation duration, but the timing of the tracheotomy was not associated with the short-term clinical endpoints’. Based on a frequentist approach, the results from this study with the updated search suggested a reduction of risk (18% for short-term mortality and 10% for VAP). The results for short-term mortality were statistically significant and those for VAP were not statistically significant. The effect estimates were very similar from the Bayesian approach but the interpretation is different.

For short-term mortality, the Bayesian approach produced posterior probabilities of more than 99% for any benefit, a small benefit (NNT<200), and a modest benefit (NNT<100). This means there is a 99% probability that for every 100 patients given an early tracheostomy, there was one additional survivor compared with late tracheostomy. For VAP, which had a non-statistically significant results from the frequentist approach, the posterior probability for any benefit was high at 94% but dropped to 78% for a small benefit and 51% for a modest benefit.

### Comparison with previous results: continuous outcomes

For the duration of mechanical ventilation and the length of ICU stay, the results from this study include new studies found in the search and some other changes the previous review. First, for a few studies the standard errors were not converted to standard deviations before consolidating all the individual study findings. Second, one of the studies^11^ in the previous review did not include a measure of variability for the outcome length of ICU stay and therefore we had to exclude it.

From a frequentist approach, following these amendments the results still showed a statistically significant reduction in the duration of mechanical ventilation and length of ICU stay. However, the reduction was much less than compared with the previous review. Again, the effect estimates were very similar to the Bayesian approach but again the interpretation is different.

For duration of mechanical ventilation and length of ICU stay, the Bayesian results are rather less impressive than were those of the frequentist analysis with lower probabilities for small and modest benefits. The posterior probabilities for extreme heterogeneity in the effect of early tracheostomy across the studies were very high for length of ICU stay which indicates the pooled estimate should be interpreted with caution.

### Implications

Scientifically, Bayesian analysis leaves considerable room for doubt; the question is not resolved and there is a case for further trials. The clinical issue is rather different as decisions have to be made on the best *current* evidence. As the point estimate is positive and ‘no evidence of effect is not evidence of no effect’, the hypothesis test is silent on what action a patient/clinician should take. This is where the Bayesian analysis proves its worth. In discussing care with the intensive care team, the clinician can now say something like; ‘there is no proof which is better, but the evidence available suggests that there is a probability of 99% that the early tracheostomy policy will reduce the risk of death in the hospital by at least one percentage point’. When speaking with the patient or family, and translating to natural frequencies, this could be described as follows: ‘There is a high chance that among 100 people in your situation, performing an early tracheostomy would mean at least one extra person would survive’.

The Bayesian analysis may also help in the design or decision to fund a further trial. The probability that the casual effect is ‘large’ with a percentage point effect of, at least one percentage point, is 99%. A frequentist trial to exclude such a difference, from a baseline of 31.5%, would require 89,898 patients across an early and late group to have 90% power at the 5% one-tailed false positive level.

Bayesian probabilities invite a consideration of preferences as they provide evidence of effects of various sizes. We think a one percentage point improvement in survival is worth known downsides of modern tracheostomy. However, it would be interesting to measure the trade-off functions for clinicians and, more importantly, their patients/family members. If most people think that a one percentage point improvement is insufficient to justify tracheostomy, then a future trial can select a larger effect size. For example, the probabilities that effect sizes exceed two and five percentage points are 96% and 62%, respectively.

### Casual pathway

The casual pathway of early tracheostomy and clinical outcomes presented in Figure 2 shows the possible mediator of VAP or other mechanisms such as reduction in sedation dose, earlier mobilisation and possibly improved mucociliary action for short-term mortality and the competing risks between short-term mortality, length of ICU stay, and duration of mechanical ventilation. Future RCTs could use this casual pathway as a basis for mediation analysis by means of structural equation modelling. Such an analysis would provide evidence for or against alternative routes from tracheostomy to outcome.

### Strengths and limitations

This study has many strengths and limitations. A strength of this study is the methods used. Bayesian meta-analysis allows for a study conclusion to be based on posterior probabilities and effect estimates which are interpreted more intuitively^33^ compared with effect estimates, CIs, and significance tests in a frequentist approach, which are often misunderstood.^34^^,^^35^ For Bayesian analysis, a choice of prior distributions for effect and heterogeneity parameters are required. Future research could use informative priors to calculate posterior probabilities as in our previous work.^36^ Our study, by repeating Deng's search identified and corrected two issues in the previous review.

The limitations of this study include differences in tracheostomy methods and definitions of early and late tracheostomy across studies. Only summary data could be extracted, so the definitions of early and late tracheostomy could not be altered and no subgroup analysis could be carried out, for example comparing before or after 9 days on a ventilator. The level of heterogeneity was very high for length of ICU stay and the results for this clinical outcome should therefore be interpreted with caution.

### Conclusions

In conclusion, the previous frequentist meta-analysis concluded that the risk of short-term mortality and VAP were not reduced for early tracheostomy compared with late tracheostomy. The frequentist results using the updated research showed a statistically significant result for short-term mortality and not for VAP. Using a Bayesian approach in this meta-analysis, we can see there is evidence that the risk of all adverse clinical outcomes is reduced for early *vs* late tracheostomy with posterior probabilities of more than 90% for any benefit and more than 75% for a small benefit apart from duration of mechanical ventilation, whereas probabilities of a modest benefit varied across outcomes from 1% to 99%.

## Authors' contributions

Study design: LQ, TV, JB, KH, TW, RL

Data analysis: LQ

Writing of the first draft of the manuscript: LG

Review and editing of the manuscript: TV, JB, KH, TW, RL

All authors reviewed the paper before submission

## Declarations of interest

The authors declare that they have no conflicts of interest.

## Funding

10.13039/501100000272National Institute for Health and Care Research (NIHR) Doctoral Research Fellowship (NIHR300606 to LQ) and the NIHR Applied Research Collaboration (ARC) West Midlands (to LQ). NIHR (16/136/87) using UK aid from the UK Government to support global health research and the NIHR ARC West Midlands (to RJL). The views expressed in this publication are those of the author(s) and not necessarily those of the NIHR or the UK government. TV is funded by the NIHR HTA (SoS, ERASER trials), MRC (CALTALYST trial), QEHB charities (acute brain injury theme) as chief investigator and MRC (Covid CNS study), i4i (LIT score in sepsis study), CDT (mechanobiology of monocyters in disease states) as co-investigator

## References

[1] Cheung N.H., Napolitano L.M. (2014). Tracheostomy: epidemiology, indications, timing, technique, and outcomes. Respir Care.

[2] Adly A., Youssef T.A., El-Begermy M.M., Younis H.M. (2018). Timing of tracheostomy in patients with prolonged endotracheal intubation: a systematic review. Eur Arch Otorhinolaryngol.

[3] Wang F., Wu Y., Bo L. (2011). The timing of tracheotomy in critically ill patients undergoing mechanical ventilation: a systematic review and meta-analysis of randomized controlled trials. Chest.

[4] Deng H., Fang Q., Chen K., Zhang X. (2021). Early versus late tracheotomy in ICU patients: a meta-analysis of randomized controlled trials. Medicine (Baltimore).

[5] Griffiths J., Barber V.S., Morgan L., Young J.D. (2005). Systematic review and meta-analysis of studies of the timing of tracheostomy in adult patients undergoing artificial ventilation. BMJ.

[6] Liu C.C., Livingstone D., Dixon E., Dort J.C. (2015). Early versus late tracheostomy: a systematic review and meta-analysis. Otolaryngol Head Neck Surg.

[7] Moher D., Hopewell S., Schulz K.F. (2012). CONSORT 2010 explanation and elaboration: updated guidelines for reporting parallel group randomised trials. Int J Surg.

[8] Röver C. (2020). Bayesian random-effects meta-analysis using the bayesmeta R package. J Stat Softw.

[9] Hamilton F.W., Lee T., Arnold D.T., Lilford R., Hemming K. (2021). Is convalescent plasma futile in COVID-19? A Bayesian re-analysis of the RECOVERY randomized controlled trial. Int J Infect Dis.

[10] Young D., Harrison D.A., Cuthbertson B.H., Rowan K., Collaborators T. (2013). Effect of early vs late tracheostomy placement on survival in patients receiving mechanical ventilation: the TracMan randomized trial. JAMA.

[11] Diaz-Prieto A., Mateu A., Gorriz M. (2014). A randomized clinical trial for the timing of tracheotomy in critically ill patients: factors precluding inclusion in a single center study. Crit Care.

[12] Cohen J. (2013).

[13] Rodriguez J.L., Steinberg S.M., Luchetti F.A., Gibbons K.J., Taheri P.A., Flint L.M. (1990). Early tracheostomy for primary airway management in the surgical critical care setting. J Br Surg.

[14] Saffle J.R., Morris S.E., Edelman L. (2002). Early tracheostomy does not improve outcome in burn patients. J Burn Care Rehabil.

[15] Bouderka M.A., Fakhir B., Bouaggad A., Hmamouchi B., Hamoudi D., Harti A. (2004). Early tracheostomy versus prolonged endotracheal intubation in severe head injury. J Trauma Acute Care Surg.

[16] Rumbak M.J., Newton M., Truncale T., Schwartz S.W., Adams J.W., Hazard P.B. (2004). A prospective, randomized, study comparing early percutaneous dilational tracheotomy to prolonged translaryngeal intubation (delayed tracheotomy) in critically ill medical patients. Crit Care Med.

[17] Barquist E.S., Amortegui J., Hallal A. (2006). Tracheostomy in ventilator dependent trauma patients: a prospective, randomized intention-to-treat study. J Trauma Acute Care Surg.

[18] Blot F., Similowski T., Trouillet J.-L. (2008). Early tracheotomy versus prolonged endotracheal intubation in unselected severely ill ICU patients. Intensive Care Med.

[19] Terragni P.P., Antonelli M., Fumagalli R. (2010). Early vs late tracheotomy for prevention of pneumonia in mechanically ventilated adult ICU patients: a randomized controlled trial. JAMA.

[20] Trouillet J.-L., Luyt C.-E., Guiguet M. (2011). Early percutaneous tracheotomy versus prolonged intubation of mechanically ventilated patients after cardiac surgery: a randomized trial. Ann Intern Med.

[21] Koch T., Hecker B., Hecker A. (2012). Early tracheostomy decreases ventilation time but has no impact on mortality of intensive care patients: a randomized study. Langenbeck's Arch Surg.

[22] Zheng Y., Sui F., Chen X.-K. (2012). Early versus late percutaneous dilational tracheostomy in critically ill patients anticipated requiring prolonged mechanical ventilation. Chin Med J.

[23] Bösel J., Schiller P., Hook Y. (2013). Stroke-related early tracheostomy versus prolonged orotracheal intubation in neurocritical care trial (SETPOINT) a randomized pilot trial. Stroke.

[24] Mohamed K.A.E., Mousa A.Y., ElSawy A.S., Saleem A.M. (2014). Early versus late percutaneous tracheostomy in critically ill adult mechanically ventilated patients. Egyptian J Chest Dis Tuberculosis.

[25] Filaire M., Tardy M.M., Richard R., Naamee A., Chadeyras J.B., Da Costa V. (2015). Prophylactic tracheotomy and lung cancer resection in patient with low predictive pulmonary function: a randomized clinical trials. Chin Clin Oncol.

[26] Karlovic Z, Vladj D, Peri M, Mihalj M, Zadro Z, Majeric -Kogler V. The impact of early percutaneous tracheotomy on reduction of the incidence of ventilator associated pneumonia and the course and outcome of ICU patients. Signa Vitae. 2018;14.

[27] Goo Z.Q., Muthusamy K.A. (2022). Early versus standard tracheostomy in ventilated patients in neurosurgical intensive care unit: A randomized controlled trial. Journal of Clinical Neuroscience.

[28] Bösel J., Niesen W-D., Salih F., Morris N.A., Ragland J.T., Gough B. (2022). Effect of early vs standard approach to tracheostomy on functional outcome at 6 months among patients with severe stroke receiving mechanical ventilation: the SETPOINT2 Randomized Clinical Trial. JAMA.

[29] Olofsson M., Pauli N., Hafsten L., Jacobsson J., Lundborg C., Brink M. (2022). TTCOV19: timing of tracheotomy in SARS-CoV-2-infected patients: a multicentre, single-blinded, randomized, controlled trial. Critical Care.

[30] Konrad F., Schreiber T., Brecht-Kraus D., Georgieff M. (1994). Mucociliary transport in ICU patients. Chest.

[31] Cavaliere F., Masieri S. (2009). Opioids and mechanical ventilation. Curr Drug Targets.

[32] Kirschenbaum L., Azzi E., Sfeir T., Tietjen P., Astiz M. (2002). Effect of continuous lateral rotational therapy on the prevalence of ventilator-associated pneumonia in patients requiring long-term ventilatory care. Crit Care Med.

[33] Spiegelhalter D.J. (2004). Incorporating Bayesian ideas into health-care evaluation. Statist Sci.

[34] Morey R.D., Hoekstra R., Rouder J.N., Lee M.D., Wagenmakers E.-J. (2016). The fallacy of placing confidence in confidence intervals. Psychon Bull Rev.

[35] Hoekstra R., Morey R.D., Rouder J.N., Wagenmakers E.-J. (2014). Robust misinterpretation of confidence intervals. Psychon Bull Rev.

[36] Hemming K., Chilton P.J., Lilford R.J., Avery A., Sheikh A. (2012). Bayesian cohort and cross-sectional analyses of the PINCER trial: a pharmacist-led intervention to reduce medication errors in primary care. PLoS One.

