# Supplementary material for: Bayesian analysis of a systematic review of early versus late tracheostomy in ICU patients
Source: Br J Anaesth. 2022 Sep 24;129(5):693–702. doi: 10.1016/j.bja.2022.08.012 (PMC9642836; doi:10.1016/j.bja.2022.08.012)
Supplement: Multimedia component 1 [file mmc1.docx]

# Supplemental material

## Posterior probability plots of heterogeneity parameters for clinical outcomes

### Short-term mortality


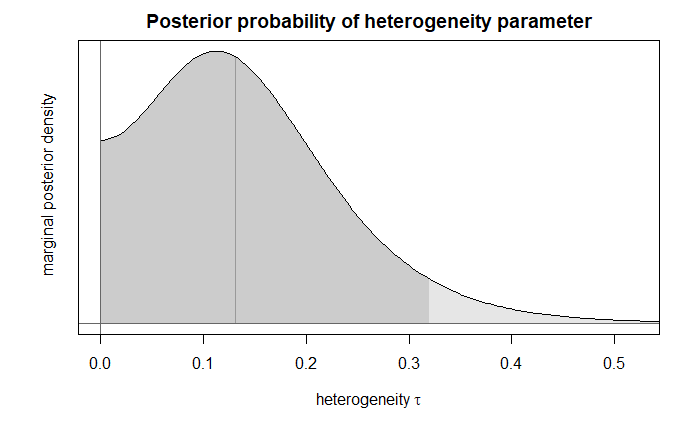


**Figure 1.** Posterior probability of heterogeneity parameter with outcome short-term mortality

### Ventilator-associated pneumonia


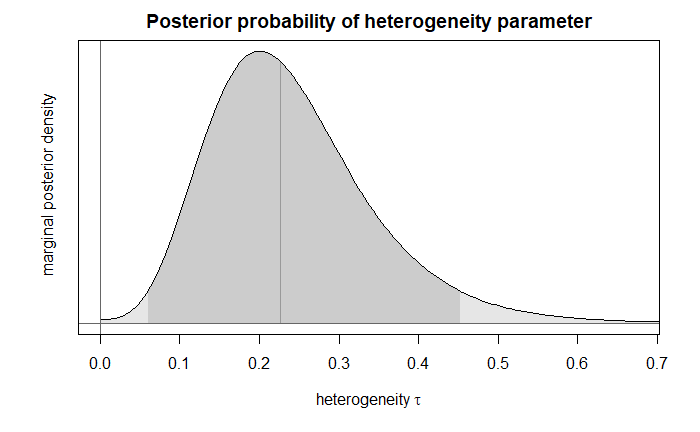


**Figure 2.** Posterior probability of heterogeneity parameter with outcome ventilator-associated pneumonia

### Duration of mechanical ventilation


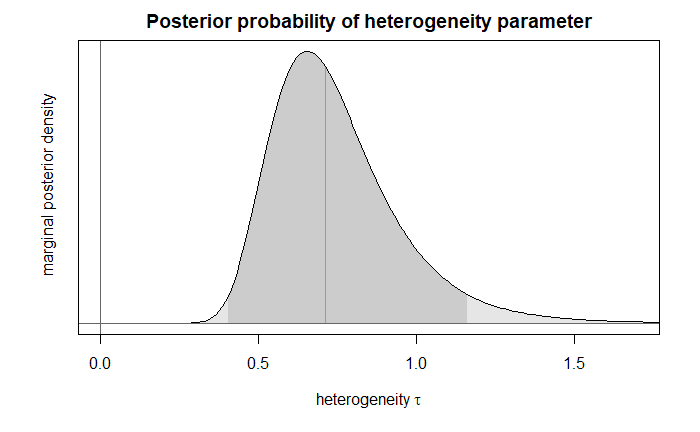


**Figure 3.** Posterior probability of heterogeneity parameter with outcome duration of mechanical ventilation

### Length of ICU stay


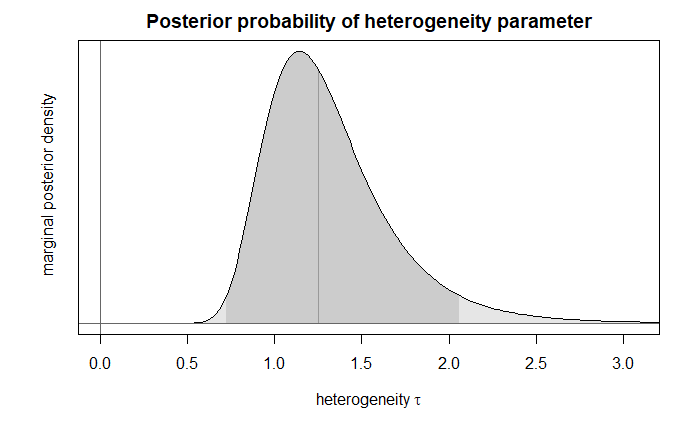


**Figure 4.** Posterior probability of heterogeneity parameter with outcome length of ICU stay

## Frequentist approach

### Short-term mortality

The pooled risk ratio for short-term mortality was 0.82 (95% CI: 0.72 to 0.94) for patients with early tracheostomy compared to late tracheostomy [*see Figure 5* ].


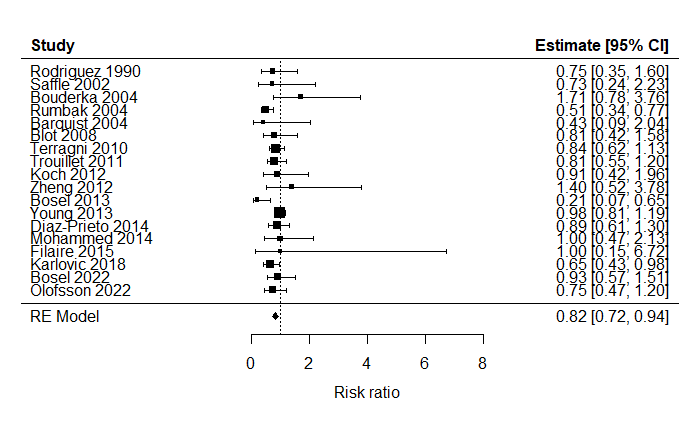


**Figure 5.** Forest plot of estimated risk ratios comparing short-term mortality in early tracheostomy vs late tracheostomy patients

### Ventilator-associated pneumonia

The pooled risk ratio for ventilator-associated pneumonia was 0. 90 (95% CI: 0.78 to 1.02) for patients with early tracheostomy compared to late tracheostomy [*see Figure 6*].


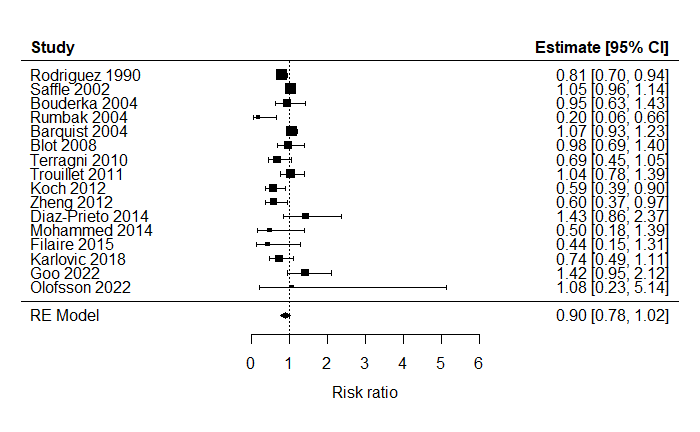


**Figure 6.** Forest plot of estimated risk ratios comparing ventilator-associated pneumonia in early tracheostomy vs late tracheostomy patients

### Duration of mechanical ventilation

The pooled standardised mean difference for duration of mechanical ventilation was -0.46 (95% CI: -0.79 to -0.13) for patients with early tracheostomy compared to late tracheostomy [*see Figure 7* ].


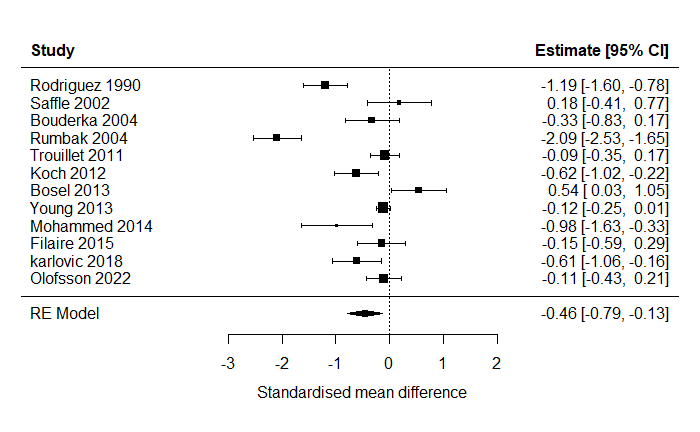


**Figure 7.** Forest plot of estimated standardised mean difference comparing duration of mechanical ventilation in early tracheostomy vs late tracheostomy patients

### Length of ICU stay

The pooled standardised mean difference for length of ICU stay was -0.73 (95% CI: -1.17 to -0.30) for patients with early tracheostomy compared to late tracheostomy [*see Figure 8*].


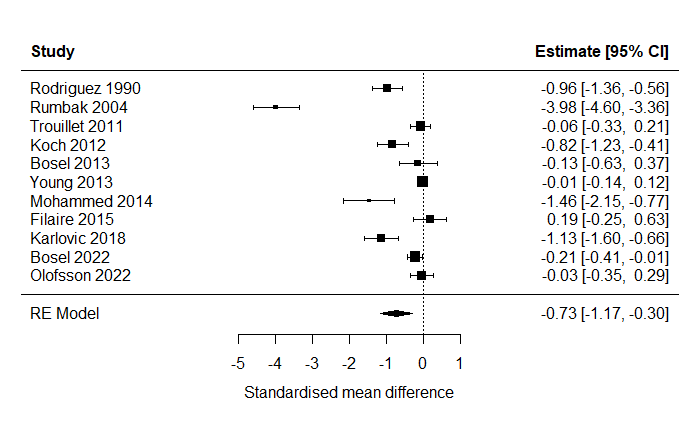


**Figure 8.** Forest plot of estimated standardised mean difference comparing length of ICU stay in early tracheostomy vs late tracheostomy patients
